# Engaging vocational dental practitioners in care of the dependent elderly: findings from a pilot project

**DOI:** 10.1038/s41415-020-1257-8

**Published:** 2020-02-28

**Authors:** Laura Beaton, Jimmy Boyle, Calum Cassie, Linda Young, Jose Marshall

**Affiliations:** 1 University of Dundee; NHS Education for Scotland, UK; 2 NHS Education for Scotland, Scotland, UK

## Abstract

**Introduction** A pilot project was developed whereby vocational dental practitioners (VDPs) became involved in the Caring for Smiles programme, visiting care homes and observing staff training.

**Aim ** The project aimed to explore VDPs' views and experiences of delivering oral healthcare in the care home environment.

**Method ** Qualitative data were collected via an online questionnaire and reflective logs. The data were analysed using thematic analysis to extract emerging themes.

**Results** Six themes emerged from the data, demonstrating that the VDPs had become aware of the general health and oral health needs of the care home population, and their role as dentists in the provision of oral care. The VDPs also had an increased awareness of Caring for Smiles.

**Discussion** The VDPs benefited from participation in the pilot; it raised their awareness but also exposed them to an environment with which many were unfamiliar. Some VDPs expressed an interest in providing oral care in care homes in the future and discussed the need for domiciliary care.

**Conclusion** The participating VDPs gained a level of experience and familiarity with older patients, Caring for Smiles and the care home environment that may improve the care they provide to their patients.

## Key points

- This paper details the findings of a pilot in which vocational dental practitioners (VDPs) became involved with Caring for Smiles, Scotland's national oral health improvement programme for dependent older people.
- The VDPs found the pilot to be beneficial, allowing them to become familiar with the care home environment and see the value of Caring for Smiles first-hand.
- VDPs became aware of barriers affecting the delivery of oral healthcare to dependent adults in the care home setting. They gained valuable skills and insights from their experience that may benefit their future patients.

## Introduction

In Scotland, the latest figures revealed that there were 31,223 long-term residents in care homes for older people.^1^ Sixty-two percent of these residents were living with dementia, 38% had a physical disability or chronic illness, 15% had visual impairment, 9% had hearing impairment and 2% had an acquired brain injury.^1^ Oral health is particularly important to dependent older people as poor oral health can affect general health and vice versa; for example, 'oral bacteria from a dental abscess or other oral infections may enter the bloodstream and cause septicaemia or blood poisoning'.^2^

The Caring for Smiles programme was developed by Scotland's National Older People's Oral Health Improvement Group in 2010 as a result of the Dental Action Plan, which identified older people as a priority group.^3^^,^^4^ Although there was an increase in dentate older adults, there remained edentulous residents within care homes who could experience problems relating to wearing and cleaning of dentures, and dentate residents often had heavily restored dentitions. These factors, in addition to the potential for care staff to encounter challenges when attempting to address residents' oral health needs, including care-resistant behaviour from residents, demonstrated that there was a need for 'tailored, evidence-based resources to promote best practice'.^4^

The Caring for Smiles Guide for Care Homes reported that there were several challenges faced when attempting to achieve and maintain good oral health in a care home environment: mouth care was considered by some to be 'distasteful'; issues regarding consent; and oral care not being considered a priority.^2^ Therefore, the Caring for Smiles programme was developed to provide training and support to care staff, in order to give them the necessary skills and knowledge so that they could provide oral healthcare and promotion to their residents. The training element of the programme is supported by guides for trainers, care homes, families and carers as part of a 'multi-faceted training and support intervention'.^2^^,^^5^^,^^6^^,^^7^ The 2012 Priority Groups Strategy noted that Caring for Smiles provided '…a valuable resource for dental professionals training care home staff in the delivery of day-to-day oral healthcare'.^8^

A recent report by the Care Inspectorate in Scotland presented the results of 145 care home inspections with a focus on the standards of care related to residents with dementia. The inspection report found that 80% of the care homes were participating in Caring for Smiles, and that staff were aware of the importance of oral care for their residents and were trained to provide this care.^9^ However, a report by the Care Quality Commission highlighted the 'growing importance for oral health in care homes' but noted that currently, in England, people in care homes are not supported 'to maintain and improve oral health'. This report found that oral health was not seen as a priority within care homes and highlighted a need for improved dental provision, improved guidance for the dental profession, and for oral health training for care home staff.^10^

In the UK, dental graduates normally complete a one-year Vocational Training (VT) or Dental Foundation Training (DFT) programme before they are eligible to work as an independent NHS practitioner in an NHS practice. The purpose of the training that covers the VT and DFT year is to 'enhance clinical and administrative competence and promote high standards'.^11^ In 2017, discussions were held between the Associate Dean for Vocational Training and the Assistant Director for Priority Groups, both within NHS Education for Scotland, about how vocational dental practitioners (VDPs) could become involved with Scotland's national oral health initiatives during their VT year and the potential benefits of their involvement. It was decided that the Caring for Smiles programme would be the initiative used for a pilot, as it was the most accessible of the priority group programmes to the VDPs, and the VT programme had had previous involvement with care home visits.

The project aimed to explore VDPs' views and experiences of delivering oral healthcare in the care home environment. Specifically, the pilot involved the participating VDPs being introduced to the Caring for Smiles programme and undertaking a series of visits to care homes in order to investigate if it would be worthwhile continuing and expanding the pilot, if changes were required for future cohorts of VDPs and what VDPs learned from their experience of the pilot.

## Method

### Sample and recruitment

An approach was made by NHS Education for Scotland's Associate Dean for VT and Assistant Director for Priority Groups to the lead for the Oral Health Improvement team in NHS Lothian's Public Dental Service (PDS) about the feasibility of conducting the pilot within the PDS. NHS Lothian's PDS agreed to become involved with the pilot and a specific VT scheme, comprising of ten VDPs based in South East Scotland, was invited to participate. Potential practical issues regarding access to care homes were resolved by the fact that the majority of VDPs within the participating scheme were already based within NHS Lothian.^11^

### Training

A 90-minute introductory session was delivered as part of the VT study programme to give VDPs the history and background of the Caring for Smiles programme. This session was delivered by NHS Lothian's Head of Oral Health Improvement and the Caring for Smiles coordinator, and outlined what the visits would entail and what the VDPs might expect when visiting care homes. The training session also provided VDPs with information regarding the role of other dental care professionals (DCPs) in the provision of care in care homes as part of Caring for Smiles, and in supporting the VDPs during their visits to care homes. The DCPs involved with Caring for Smiles include: a dental hygienist, an oral health promoter and dental health support workers. In addition, hygienists and therapists are used by the PDS to carry out some treatment within care homes, including scale and polish and placing dressings.

All participating VDPs visited care homes with the Caring for Smiles team for two non-consecutive days. Where possible, one of these days included observing part of a Caring for Smiles training session for care home staff. During these visits, the VDPs were acting in the capacity of a member of the Caring for Smiles team, which involved carrying out oral health assessments and delivering preventive treatment for residents.

### Data collection

VDPs completed an online qualitative questionnaire asking about their involvement with the pilot, their thoughts about the provision of oral healthcare for elderly patients and their role as dentists in this provision. Data from VDPs' reflective logs, kept as part of their training e-portfolios, were also included in the analysis.

### Data analysis

All responses were anonymised before data analysis was conducted. The reflective logs and responses to the questionnaire were analysed using thematic analysis, which is a method for 'identifying, analysing, organising, describing and reporting themes' in qualitative data.^12^ There are six stages to thematic analysis: familiarisation with the data; generating initial coding; searching for themes; reviewing themes; defining and naming themes; and producing the report.^,^^12^^,^^13^

## Results

All ten VDPs that were invited to participate agreed to take part in the pilot.

Thematic analysis of the qualitative data from both the questionnaire and reflective logs revealed six main themes concerning the VDPs' experiences and reflections on their involvement with the Caring for Smiles programme, the pilot and their roles as dentists in the provision of oral care for care home residents. These will now be discussed.

### Professional roles

VDPs raised the topic of professional roles in Caring for Smiles, including the role of care home staff and the role of the dentist. They suggested that dentists were key in helping older patients maintain or improve their oral health, a role that should also include visiting care homes to deliver oral care.

'*As a dentist I see my role as to act if patients in care homes who are registered with me require treatment. It is important to have a good relationship with care staff and communicate regularly to see if there are any problems*.'

The VDPs saw care home staff as ideally placed to deliver oral care to their residents and 'to be enforcing oral hygiene regimes daily'. However, the VDPs noted that there needed to be consistent training for care home staff and that oral health needed to be seen as a priority in the care homes.

VDPs also stated that dental healthcare professionals should visit care homes, not only to deliver Caring for Smiles training but also to support care home staff to maintain the oral hygiene of the residents. VDPs also noted the importance of communication between all professionals involved in the healthcare of the care home residents, stressing the need to share information and avoid conflicting advice.

### Challenges of delivering Caring for Smiles

Taking part in the pilot had made the VDPs aware of the challenges associated with Caring for Smiles. These challenges were varied. Some VDPs raised the issues of funding and the necessary paperwork required to ensure that oral health was not 'skipped'.

'*Trying to maintain good oral hygiene in the frail and elderly is challenging for a dentist, never mind someone who doesn't routinely work in dentistry*.'

Other VDPs highlighted that training of care home staff was crucial for the success of Caring for Smiles and for the maintenance of good oral health in the care home residents. One VDP noted that they now appreciated that care home staff may not have any prior understanding about how to deliver oral health advice and support to their residents without training. However, there were challenges associated with this, namely the compliance of care homes and accessing staff to train:

'*Care homes sometimes have high turnover of staff and rely on agency - making training all members of staff difficult*.'

In addition, effective patient management posed a challenge to the delivery of Caring for Smiles: some patients required more time to be seen, and others were uncooperative or had communication problems.

*'(One) case mainly emphasised the difficulty in gaining consent as the patient was completely uncooperative, but it also showed the difficulty in communication with a dentist to be arranged to extract this tooth for the patient… the overall management for these patients can be challenging*.'

Participants also expressed frustration at what they perceived to be the inefficiency of the Caring for Smiles visits, as they often necessitated travel between care homes and waiting on patients:

'*I spent 80% of my day driving in the car from care home to care home (three care homes in total) and waiting on the patients to be ready to be seen (eating their breakfast, lunch etc) than actually looking in patients' mouths*.'

### Oral health status

Prior to their involvement with Caring for Smiles, VDPs were hitherto largely unaware of the oral health status of dependent older people. They expressed frustration and shock at what they had observed during their visits to the care homes, particularly the lack of routine dental care available to the residents. One VDP stated that they were disappointed and upset by the oral health status of patients they interacted with during their visit. In addition, their experiences of the pilot allowed the VDPs to see the benefits of Caring for Smiles, as it helped to address this unmet oral health need.

'*I was shocked at the fact that routine dental care does not exist in care homes… some patients had not visited/been visited by a dentist in years… it was clear dental treatment and frequent examinations were required*.'

One VDP acknowledged that they now appreciated the difficulties involved in providing oral care in care homes. Another observed that most residents had dentures, which were difficult to manage. They also expressed concern about the potential difficulties in managing more complex dental treatment (for example, crown and bridgework or implants) that future care home residents may have.

### General health

Alongside an increase in awareness of the oral health of dependent older people, the VDPs also became more aware of the general health of the residents, as well as gaining understanding in how their health affected the provision of oral care, in particular for patients with dementia or those who had difficulties communicating.

'*This age group presents a particular challenge as many of these patients suffer from dementia and often cannot communicate when something is wrong with their mouths*.'

In addition, VDPs acknowledged that this population were affected by other health concerns and stressed the need to maintain good nutrition and dentition, particularly when residents may be suffering from other medical conditions that affected their oral health. Furthermore, the VDPs reflected that this was an 'ageing population' and that, in the future, increasingly more people would require care in a care home setting.

### Benefits of Caring for Smiles

VDPs participating in the pilot also gained insight into Caring for Smiles, particularly the benefits of the programme and the value to the care home residents. The VDPs recognised the unmet need experienced by dependent older people, which appeared to make them appreciate the need for Caring for Smiles.

'*I noticed from my time in the care homes I visited that there was a very great need for dental treatment in all of them.*'

When asked how oral health care could best be delivered to the dependent elderly, one participant specifically named Caring for Smiles and participants praised the programme: '*I think the Caring for Smiles programme is excellent and it is enabling monitoring of oral health to some degree*.' Furthermore, one participant noted the positive influence that Caring for Smiles has on a care home's Care Inspectorate inspection, citing it as a reason for care homes to become involved with Caring for Smiles.

### Benefits of pilot to VDPs

VDPs discussed how they had enjoyed their experiences of Caring for Smiles; some highlighted that they had enjoyed learning something new, while others enjoyed the hands-on experience interacting with the residents, with one stating: '*I met a lady who was 105! I felt very humble*.' Other participants referred to their experiences as 'eye-opening' and how their involvement allowed them the opportunity to gain familiarity with the care home environment and gain a better understanding of patient need.

'*I don't know that it will ever be a comfortable environment to be in a care home; however, I am very grateful for the opportunity to get over the initial shock and anxiety of going into one in this controlled setting.*'

Other VDPs reflected that learning about and getting a first-hand experience of Caring for Smiles was beneficial to their careers as dentists, particularly if they were dealing with dependent elderly patients in the future.

'*It was a great experience and one which is very beneficial especially at our early stages in this career. It allows for exposure to this environment and group of patients early so that in the future, should we have to manage the elderly population, which is most likely, we will feel more equipped and less intimidated to treat them.*'

## Discussion

The feedback from the questionnaire and the reflective logs suggested that the VDPs see the value of the Caring for Smiles programme and the benefits to care home residents. Their experience in the pilot had raised their awareness of the oral health status of this patient group and the challenges faced by Caring for Smiles teams and care home staff while attempting to address the oral health needs of care home residents.

The reflective logs highlighted that the VDPs had benefited from taking part in the pilot. Not only had the experience raised their awareness, it had also provided them with the opportunity to become familiar with the care home environment, which some noted would serve them well in their future careers, particularly as some expressed initial discomfort and unfamiliarity with this environment. In the logs, some expressed an interest in carrying out work in care homes in the future and discussed the need for domiciliary care, given the difficulties care residents experience regarding access to oral care. Indeed, Scotland's Oral Health Improvement Plan has stressed the importance of meeting the needs of an ageing population, including improved domiciliary care provision.^14^ Specifically, the Oral Health Improvement Plan will 'introduce arrangements to enable accredited GDPs to provide care in care homes' with the intention of reducing the burden on the PDS by supporting GDPs to work alongside care home staff to provide oral care.^14^

As mentioned in the introduction, the pilot aimed to investigate if it was worthwhile to continue with a second cohort of VDPs and what adjustments should be made following the feedback from the first cohort. While the pilot was a beneficial experience for the VDPs, there are still some issues to be addressed before it is repeated in the next training year. As noted above, VDPs commented that they had enjoyed the more hands-on elements of their experience, rather than observing training sessions. VDPs also reported being frustrated at the inefficiency of their visits, wishing they could see more patients and visit one care home per day, rather than travelling between them. However, the logistical and practical constraints that can affect the delivery of Caring for Smiles are often out of the control of the Caring for Smiles team and may not be fully appreciated by the VDPs. Therefore, for the next training year, the VDPs will receive teaching sessions about capacity, consent in the elderly and the delivery of treatment by a dentist in the care home, so that the VDPs can see how this complements Caring for Smiles and so that they appreciate the difference between the two roles. In addition, when the Caring for Smiles team were asked about the scheduling of visits and why the VDPs were travelling between care homes, it emerged that their visits had been deliberately scheduled in this way, so that the VDPs could experience a variety of care homes and residents. This point has since been included in the introductory session for the next cohort of VDPs.

## Conclusion

In conclusion, the pilot succeeded in raising awareness of the oral health needs of care home residents and provided the VDPs with a unique opportunity to engage with a different patient group in a non-clinical environment. The VDPs taking part in the pilot now have a level of experience and familiarity with Caring for Smiles and the care home environment that their peers may not have. In addition, experience engaging with older people at an early stage in dentists' careers is likely to be beneficial to the older patients that they will treat in the future. This is particularly relevant given that improved domiciliary care provision is listed as an action in the new Oral Health Improvement Plan to meet the needs of an ageing population.^14^ By participating in the pilot, the VDPs gained valuable skills and insights, enabling them to provide improved care to patients in general practice who, although they may not be residents of a care home, may share many of the same patient management challenges. It is intended that this project can continue beyond a second cohort, expanding involvement to include other VDP schemes and other NHS Health Boards and, potentially, Dental Therapy Vocational Trainees. Furthermore, other national oral health initiatives for priority groups may also be included.
